# (*Z*)-5-Benzene­carbothioyl-1,11-dimethyl-6-phenyl-5*H*-dibenzo[*d*,*f*][1,3]diazepine

**DOI:** 10.1107/S1600536811000092

**Published:** 2011-01-12

**Authors:** Jia-Xin Zhang, Seik Weng Ng

**Affiliations:** a College of Chemistry, Beijing Normal University, Beijing 100875, People’s Republic of China; b Department of Chemistry, University of Malaya, 50603 Kuala Lumpur, Malaysia

## Abstract

The seven-membered ring in the title compound, C_28_H_22_N_2_S, has a two-coordinate N atom as well as a three-coordinate N atom. The ring adopts a boat-shaped conformation with two C atoms of one methyl­phenyl ring as the stern and the three-coordinate N atom as the prow. The *N*,*N*-dimethyl­ethane­thio­amide fragment is nearly planar (r.m.s. deviation = 0.049 Å); the phenyl ring of the benzene­carbothioyl unit connected to the three-coordinate N atom is aligned at 83.72 (4)° with respect to the mean plane of this fragment. Weak inter­molecular C—H⋯S hydrogen bonding is present in the crystal structure.

## Related literature

For background to the synthesis of thio­amides by the reaction of 1,1′-binaphthyl-2,2′-diamine with acyl chlorides and phospho­rus penta­sulfide, see: Shi *et al.* (2004 ▶).
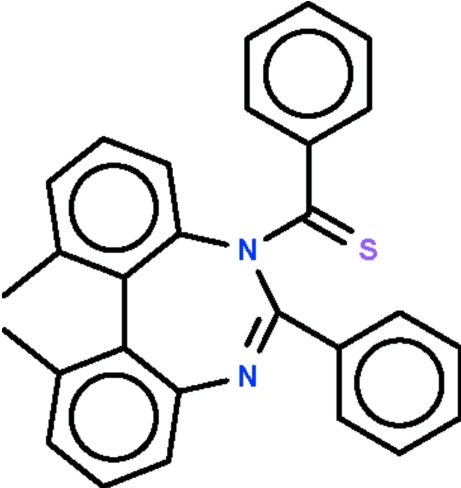


## Experimental

### 

#### Crystal data

C_28_H_22_N_2_S
*M* *_r_* = 418.54
Orthorhombic, 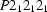

*a* = 8.7665 (18) Å
*b* = 11.605 (3) Å
*c* = 21.392 (5) Å
*V* = 2176.4 (8) Å^3^
*Z* = 4
Mo *K*α radiation
μ = 0.17 mm^−1^
*T* = 113 K
0.26 × 0.22 × 0.20 mm

#### Data collection

Rigaku Saturn CCD area-detector diffractometer
Absorption correction: multi-scan (*CrystalClear*; Rigaku/MSC, 2005 ▶) *T*_min_ = 0.958, *T*_max_ = 0.967
21933 measured reflections
5176 independent reflections
4527 reflections with *I* > 2σ(*I*)
*R*_int_ = 0.050

#### Refinement

*R*[*F* ^2^ > 2σ(*F* ^2^)] = 0.039
*wR*(*F* ^2^) = 0.085
*S* = 0.99
5176 reflections
282 parameters
H-atom parameters constrained
Δρ_max_ = 0.19 e Å^−3^
Δρ_min_ = −0.23 e Å^−3^
Absolute structure: Flack (1983 ▶), 2775 Friedel pairs
Flack parameter: −0.03 (6)

### 

Data collection: *CrystalClear* (Rigaku/MSC, 2005 ▶); cell refinement: *CrystalClear*; data reduction: *CrystalClear*; program(s) used to solve structure: *SHELXS97* (Sheldrick, 2008 ▶); program(s) used to refine structure: *SHELXL97* (Sheldrick, 2008 ▶); molecular graphics: *X-SEED* (Barbour, 2001 ▶); software used to prepare material for publication: *publCIF* (Westrip, 2010 ▶).

## Supplementary Material

Crystal structure: contains datablocks global, I. DOI: 10.1107/S1600536811000092/xu5136sup1.cif

Structure factors: contains datablocks I. DOI: 10.1107/S1600536811000092/xu5136Isup2.hkl

Additional supplementary materials:  crystallographic information; 3D view; checkCIF report

## Figures and Tables

**Table 1:** Hydrogen-bond geometry (Å, °)

*D*—H⋯*A*	*D*—H	H⋯*A*	*D*⋯*A*	*D*—H⋯*A*
C3—H3⋯S1^i^	0.95	2.86	3.7216 (18)	152

Symmetry code: (i) 
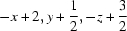
.

**Fig. 1.**
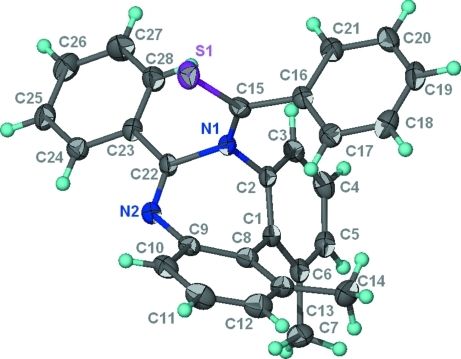
Thermal ellipsoid plot (Barbour, 2001) of C28H22N2S at the 70% probability level; hydrogen atoms are drawn as spheres of arbitrary radius.

## supplementary crystallographic information

### Comment

The two amino groups of 1,1'-binaphthyl-2,2'-diamine undergo condensation with acyl chlorides and phosphorus pentasulfide to yield bis(thioamides) (Shi *et al.*, 2004). The present study represents a variation of this reaction by reacting a biphenyl dibenzamide derivative directly with phosphorus pentasulfide. The resulting compound (Scheme I) has only one carbonyl fragment replaced by a thiocarbonyl fragment as the other fragment has undergone cyclization (Scheme I). The compound features a seven-membered ring, with the two nitrogen atoms constituting members of a seven-membered ring.

### Experimental

*N*,*N*'-(6,6'-Dimethylbiphenyl-2,2'-diyl)dibenzamide (210 mg, 0.50 mmol) and phosphorus pentasulfide (111 mg, 0.5 mmol) in pyridine (2 ml) were heated at 393 K for 10 h. To the product was added 10% sodium hydroxide to a pH of about 10. The organic compound was extracted with ethyl acetate (3 *x* 20 ml). The organic phase was dried over sodium sulfate. The solvent was removed under reduced pressure and the residue was purified by column chromatography (eluent: ethyl acetate/petroleum ether 1/20) to give the title compound (46.8 mg, 20%) as a yellow solid. Single crystals were obtained upon recrystallized from a dichloromethane-hexane mixture.

### Refinement

Carbon-bound H-atoms were placed in calculated positions (C–H 0.95–0.98 Å) and were included in the refinement in the riding model approximation, with *U*_iso_(H) set to 1.2–1.5*U*_eq_(C).

### Crystal data

**Table:**

C_28_H_22_N_2_S	*F*(000) = 880
*M**_r_* = 418.54	*D*_x_ = 1.277 Mg m^−^^3^
Orthorhombic, *P*2_1_2_1_2_1_	Mo *K*α radiation, λ = 0.71070 Å
Hall symbol: P 2ac 2ab	Cell parameters from 7653 reflections
*a* = 8.7665 (18) Å	θ = 1.9–27.9°
*b* = 11.605 (3) Å	µ = 0.17 mm^−^^1^
*c* = 21.392 (5) Å	*T* = 113 K
*V* = 2176.4 (8) Å^3^	Block, yellow
*Z* = 4	0.26 × 0.22 × 0.20 mm

### Data collection

**Table:**

Rigaku Saturn CCD area-detector diffractometer	5176 independent reflections
Radiation source: rotating anode	4527 reflections with *I* > 2σ(*I*)
confocal	*R*_int_ = 0.050
Detector resolution: 7.31 pixels mm^-1^	θ_max_ = 27.9°, θ_min_ = 1.9°
ω and φ scans	*h* = −11→9
Absorption correction: multi-scan (*CrystalClear*; Rigaku/MSC, 2005)	*k* = −15→15
*T*_min_ = 0.958, *T*_max_ = 0.967	*l* = −28→28
21933 measured reflections	

### Refinement

**Table:**

Refinement on *F*^2^	Secondary atom site location: difference Fourier map
Least-squares matrix: full	Hydrogen site location: inferred from neighbouring sites
*R*[*F*^2^ > 2σ(*F*^2^)] = 0.039	H-atom parameters constrained
*wR*(*F*^2^) = 0.085	*w* = 1/[σ^2^(*F*_o_^2^) + (0.0493*P*)^2^] where *P* = (*F*_o_^2^ + 2*F*_c_^2^)/3
*S* = 0.99	(Δ/σ)_max_ = 0.001
5176 reflections	Δρ_max_ = 0.19 e Å^−^^3^
282 parameters	Δρ_min_ = −0.23 e Å^−^^3^
0 restraints	Absolute structure: Flack (1983), 2775 Friedel pairs
Primary atom site location: structure-invariant direct methods	Flack parameter: −0.03 (6)

### Fractional atomic coordinates and isotropic or equivalent isotropic displacement parameters (Å^2^)

**Table:**

	*x*	*y*	*z*	*U*_iso_*/*U*_eq_	
S1	1.09723 (5)	0.47544 (4)	0.76793 (2)	0.02825 (11)	
N1	0.95131 (14)	0.56476 (11)	0.67231 (6)	0.0178 (3)	
N2	0.76743 (15)	0.41503 (11)	0.66647 (6)	0.0200 (3)	
C1	0.88897 (18)	0.55478 (13)	0.56264 (7)	0.0187 (3)	
C2	0.92563 (17)	0.62439 (13)	0.61387 (7)	0.0178 (3)	
C3	0.91869 (17)	0.74326 (13)	0.61224 (7)	0.0209 (3)	
H3	0.9482	0.7880	0.6474	0.025*	
C4	0.86735 (18)	0.79547 (14)	0.55772 (8)	0.0240 (4)	
H4	0.8658	0.8771	0.5546	0.029*	
C5	0.81855 (18)	0.72897 (15)	0.50809 (8)	0.0237 (4)	
H5	0.7770	0.7660	0.4723	0.028*	
C6	0.82841 (18)	0.60889 (14)	0.50890 (7)	0.0214 (3)	
C7	0.7658 (2)	0.54265 (15)	0.45397 (8)	0.0288 (4)	
H7A	0.6619	0.5690	0.4449	0.043*	
H7B	0.7641	0.4602	0.4640	0.043*	
H7C	0.8307	0.5556	0.4173	0.043*	
C8	0.91836 (18)	0.42900 (13)	0.56802 (7)	0.0194 (3)	
C9	0.85781 (17)	0.36525 (13)	0.61850 (7)	0.0197 (3)	
C10	0.88359 (19)	0.24726 (13)	0.62336 (8)	0.0248 (4)	
H10	0.8367	0.2042	0.6559	0.030*	
C11	0.9777 (2)	0.19306 (15)	0.58068 (8)	0.0293 (4)	
H11	0.9968	0.1128	0.5843	0.035*	
C12	1.0442 (2)	0.25546 (14)	0.53263 (8)	0.0270 (4)	
H12	1.1099	0.2175	0.5039	0.032*	
C13	1.01628 (18)	0.37322 (14)	0.52574 (7)	0.0221 (3)	
C14	1.1039 (2)	0.43886 (15)	0.47629 (8)	0.0291 (4)	
H14A	1.1978	0.3970	0.4660	0.044*	
H14B	1.1298	0.5156	0.4921	0.044*	
H14C	1.0409	0.4464	0.4387	0.044*	
C15	1.08502 (18)	0.54891 (12)	0.70153 (7)	0.0193 (3)	
C16	1.22481 (18)	0.59577 (13)	0.67023 (7)	0.0192 (3)	
C17	1.29579 (18)	0.52874 (14)	0.62485 (7)	0.0228 (3)	
H17	1.2494	0.4592	0.6111	0.027*	
C18	1.43445 (19)	0.56342 (15)	0.59970 (8)	0.0271 (4)	
H18	1.4845	0.5166	0.5696	0.032*	
C19	1.49930 (19)	0.66606 (16)	0.61859 (8)	0.0272 (4)	
H19	1.5942	0.6898	0.6015	0.033*	
C20	1.4268 (2)	0.73423 (15)	0.66218 (8)	0.0284 (4)	
H20A	1.4712	0.8055	0.6743	0.034*	
C21	1.28956 (19)	0.69967 (14)	0.68849 (8)	0.0249 (4)	
H21A	1.2403	0.7467	0.7187	0.030*	
C22	0.81179 (17)	0.50720 (13)	0.69330 (7)	0.0196 (3)	
C23	0.72239 (18)	0.56611 (13)	0.74184 (7)	0.0200 (3)	
C24	0.58133 (17)	0.52104 (14)	0.75993 (7)	0.0226 (3)	
H24	0.5459	0.4509	0.7423	0.027*	
C25	0.4935 (2)	0.57849 (15)	0.80349 (8)	0.0269 (4)	
H25	0.3987	0.5467	0.8164	0.032*	
C26	0.5424 (2)	0.68208 (16)	0.82853 (8)	0.0312 (4)	
H26	0.4804	0.7219	0.8579	0.037*	
C27	0.6819 (2)	0.72744 (16)	0.81069 (9)	0.0348 (4)	
H27	0.7159	0.7983	0.8280	0.042*	
C28	0.7718 (2)	0.66969 (15)	0.76768 (8)	0.0279 (4)	
H28	0.8677	0.7009	0.7557	0.033*	

### Atomic displacement parameters (Å^2^)

**Table:**

	*U*^11^	*U*^22^	*U*^33^	*U*^12^	*U*^13^	*U*^23^
S1	0.0262 (2)	0.0317 (2)	0.0269 (2)	−0.00369 (19)	−0.00223 (19)	0.01130 (18)
N1	0.0185 (7)	0.0179 (6)	0.0171 (6)	−0.0035 (5)	0.0025 (5)	0.0004 (5)
N2	0.0197 (7)	0.0204 (7)	0.0198 (6)	−0.0026 (5)	0.0019 (6)	0.0006 (5)
C1	0.0164 (8)	0.0212 (8)	0.0183 (7)	−0.0023 (6)	0.0034 (6)	0.0007 (6)
C2	0.0147 (8)	0.0211 (8)	0.0177 (7)	−0.0017 (6)	0.0023 (6)	0.0025 (6)
C3	0.0175 (8)	0.0197 (7)	0.0256 (8)	−0.0021 (6)	0.0024 (7)	−0.0020 (6)
C4	0.0205 (9)	0.0187 (8)	0.0328 (9)	0.0001 (6)	0.0026 (7)	0.0050 (7)
C5	0.0193 (9)	0.0281 (9)	0.0236 (8)	−0.0005 (7)	0.0018 (7)	0.0069 (7)
C6	0.0184 (8)	0.0241 (8)	0.0215 (8)	−0.0019 (6)	0.0028 (7)	0.0036 (7)
C7	0.0318 (9)	0.0323 (10)	0.0223 (8)	−0.0020 (8)	−0.0047 (8)	0.0015 (7)
C8	0.0181 (8)	0.0200 (7)	0.0202 (7)	−0.0006 (6)	−0.0016 (7)	−0.0020 (6)
C9	0.0163 (8)	0.0213 (8)	0.0214 (8)	−0.0020 (6)	−0.0018 (6)	−0.0025 (7)
C10	0.0255 (9)	0.0204 (8)	0.0285 (8)	−0.0034 (7)	−0.0046 (7)	0.0006 (7)
C11	0.0301 (9)	0.0186 (8)	0.0391 (10)	0.0029 (7)	−0.0038 (8)	−0.0032 (7)
C12	0.0239 (8)	0.0270 (9)	0.0300 (9)	0.0048 (7)	−0.0011 (7)	−0.0068 (7)
C13	0.0208 (8)	0.0240 (8)	0.0214 (8)	−0.0006 (7)	−0.0011 (7)	−0.0029 (7)
C14	0.0282 (9)	0.0339 (9)	0.0253 (8)	0.0005 (8)	0.0068 (8)	−0.0031 (7)
C15	0.0191 (8)	0.0158 (7)	0.0229 (8)	−0.0012 (6)	0.0002 (7)	−0.0030 (6)
C16	0.0161 (8)	0.0207 (7)	0.0208 (7)	0.0002 (6)	−0.0024 (7)	0.0054 (6)
C17	0.0238 (8)	0.0217 (8)	0.0230 (8)	0.0002 (7)	−0.0016 (7)	0.0035 (7)
C18	0.0269 (9)	0.0321 (9)	0.0223 (8)	0.0078 (7)	0.0042 (7)	0.0056 (7)
C19	0.0186 (8)	0.0374 (10)	0.0257 (9)	−0.0021 (7)	0.0009 (7)	0.0106 (8)
C20	0.0284 (10)	0.0289 (8)	0.0280 (9)	−0.0103 (7)	−0.0028 (8)	0.0061 (7)
C21	0.0252 (9)	0.0239 (8)	0.0256 (8)	−0.0021 (7)	0.0011 (7)	0.0000 (7)
C22	0.0176 (8)	0.0219 (8)	0.0193 (7)	−0.0036 (6)	0.0020 (6)	0.0032 (6)
C23	0.0190 (8)	0.0217 (7)	0.0192 (7)	0.0003 (6)	0.0019 (7)	0.0024 (6)
C24	0.0217 (8)	0.0244 (7)	0.0217 (7)	−0.0028 (7)	−0.0017 (7)	0.0025 (7)
C25	0.0189 (8)	0.0394 (10)	0.0226 (8)	−0.0012 (7)	0.0036 (7)	0.0034 (7)
C26	0.0304 (10)	0.0368 (9)	0.0265 (9)	0.0057 (8)	0.0071 (8)	−0.0022 (8)
C27	0.0392 (11)	0.0298 (10)	0.0353 (10)	−0.0039 (8)	0.0088 (9)	−0.0118 (8)
C28	0.0267 (9)	0.0279 (9)	0.0290 (9)	−0.0056 (7)	0.0070 (8)	−0.0052 (8)

### Geometric parameters (Å, °)

**Table:**

S1—C15	1.6601 (16)	C13—C14	1.513 (2)
N1—C15	1.341 (2)	C14—H14A	0.9800
N1—C2	1.4465 (19)	C14—H14B	0.9800
N1—C22	1.4642 (19)	C14—H14C	0.9800
N2—C22	1.275 (2)	C15—C16	1.499 (2)
N2—C9	1.419 (2)	C16—C21	1.389 (2)
C1—C2	1.399 (2)	C16—C17	1.391 (2)
C1—C6	1.413 (2)	C17—C18	1.389 (2)
C1—C8	1.487 (2)	C17—H17	0.9500
C2—C3	1.381 (2)	C18—C19	1.380 (2)
C3—C4	1.389 (2)	C18—H18	0.9500
C3—H3	0.9500	C19—C20	1.378 (2)
C4—C5	1.381 (2)	C19—H19	0.9500
C4—H4	0.9500	C20—C21	1.388 (2)
C5—C6	1.396 (2)	C20—H20A	0.9500
C5—H5	0.9500	C21—H21A	0.9500
C6—C7	1.508 (2)	C22—C23	1.470 (2)
C7—H7A	0.9800	C23—C24	1.397 (2)
C7—H7B	0.9800	C23—C28	1.392 (2)
C7—H7C	0.9800	C24—C25	1.380 (2)
C8—C13	1.405 (2)	C24—H24	0.9500
C8—C9	1.413 (2)	C25—C26	1.384 (3)
C9—C10	1.392 (2)	C25—H25	0.9500
C10—C11	1.382 (2)	C26—C27	1.385 (3)
C10—H10	0.9500	C26—H26	0.9500
C11—C12	1.386 (2)	C27—C28	1.385 (2)
C11—H11	0.9500	C27—H27	0.9500
C12—C13	1.396 (2)	C28—H28	0.9500
C12—H12	0.9500		
			
C15—N1—C2	127.19 (12)	C13—C14—H14B	109.5
C15—N1—C22	121.64 (12)	H14A—C14—H14B	109.5
C2—N1—C22	110.69 (12)	C13—C14—H14C	109.5
C22—N2—C9	119.79 (13)	H14A—C14—H14C	109.5
C2—C1—C6	117.83 (14)	H14B—C14—H14C	109.5
C2—C1—C8	117.81 (13)	N1—C15—C16	117.18 (13)
C6—C1—C8	124.35 (14)	N1—C15—S1	121.71 (11)
C3—C2—C1	123.16 (14)	C16—C15—S1	121.06 (12)
C3—C2—N1	120.42 (14)	C21—C16—C17	119.94 (15)
C1—C2—N1	115.88 (13)	C21—C16—C15	121.58 (14)
C2—C3—C4	118.10 (15)	C17—C16—C15	118.34 (14)
C2—C3—H3	120.9	C18—C17—C16	119.99 (16)
C4—C3—H3	120.9	C18—C17—H17	120.0
C5—C4—C3	120.14 (15)	C16—C17—H17	120.0
C5—C4—H4	119.9	C19—C18—C17	119.80 (16)
C3—C4—H4	119.9	C19—C18—H18	120.1
C4—C5—C6	121.95 (16)	C17—C18—H18	120.1
C4—C5—H5	119.0	C18—C19—C20	120.25 (16)
C6—C5—H5	119.0	C18—C19—H19	119.9
C5—C6—C1	118.48 (15)	C20—C19—H19	119.9
C5—C6—C7	118.46 (15)	C19—C20—C21	120.55 (16)
C1—C6—C7	122.97 (14)	C19—C20—H20A	119.7
C6—C7—H7A	109.5	C21—C20—H20A	119.7
C6—C7—H7B	109.5	C16—C21—C20	119.43 (16)
H7A—C7—H7B	109.5	C16—C21—H21A	120.3
C6—C7—H7C	109.5	C20—C21—H21A	120.3
H7A—C7—H7C	109.5	N2—C22—N1	119.97 (14)
H7B—C7—H7C	109.5	N2—C22—C23	123.08 (14)
C13—C8—C9	118.71 (14)	N1—C22—C23	116.74 (13)
C13—C8—C1	120.56 (14)	C24—C23—C28	119.25 (15)
C9—C8—C1	120.55 (14)	C24—C23—C22	119.57 (14)
C10—C9—C8	120.75 (15)	C28—C23—C22	121.08 (14)
C10—C9—N2	115.93 (15)	C25—C24—C23	119.98 (15)
C8—C9—N2	123.32 (14)	C25—C24—H24	120.0
C11—C10—C9	119.71 (16)	C23—C24—H24	120.0
C11—C10—H10	120.1	C24—C25—C26	120.54 (16)
C9—C10—H10	120.1	C24—C25—H25	119.7
C10—C11—C12	120.21 (16)	C26—C25—H25	119.7
C10—C11—H11	119.9	C25—C26—C27	119.79 (17)
C12—C11—H11	119.9	C25—C26—H26	120.1
C11—C12—C13	121.07 (16)	C27—C26—H26	120.1
C11—C12—H12	119.5	C28—C27—C26	120.12 (17)
C13—C12—H12	119.5	C28—C27—H27	119.9
C12—C13—C8	119.35 (15)	C26—C27—H27	119.9
C12—C13—C14	118.53 (15)	C27—C28—C23	120.30 (16)
C8—C13—C14	121.89 (14)	C27—C28—H28	119.8
C13—C14—H14A	109.5	C23—C28—H28	119.8
			
C6—C1—C2—C3	−6.1 (2)	C9—C8—C13—C14	−170.84 (15)
C8—C1—C2—C3	173.00 (15)	C1—C8—C13—C14	4.3 (2)
C6—C1—C2—N1	165.46 (13)	C2—N1—C15—C16	−1.2 (2)
C8—C1—C2—N1	−15.4 (2)	C22—N1—C15—C16	170.10 (13)
C15—N1—C2—C3	−80.0 (2)	C2—N1—C15—S1	−178.48 (11)
C22—N1—C2—C3	107.88 (16)	C22—N1—C15—S1	−7.2 (2)
C15—N1—C2—C1	108.11 (17)	N1—C15—C16—C21	99.88 (18)
C22—N1—C2—C1	−63.97 (16)	S1—C15—C16—C21	−82.82 (18)
C1—C2—C3—C4	2.5 (2)	N1—C15—C16—C17	−84.55 (18)
N1—C2—C3—C4	−168.78 (13)	S1—C15—C16—C17	92.75 (16)
C2—C3—C4—C5	2.9 (2)	C21—C16—C17—C18	2.6 (2)
C3—C4—C5—C6	−4.4 (2)	C15—C16—C17—C18	−173.01 (14)
C4—C5—C6—C1	0.6 (2)	C16—C17—C18—C19	−1.8 (2)
C4—C5—C6—C7	177.21 (15)	C17—C18—C19—C20	−0.1 (3)
C2—C1—C6—C5	4.5 (2)	C18—C19—C20—C21	1.2 (3)
C8—C1—C6—C5	−174.62 (15)	C17—C16—C21—C20	−1.5 (2)
C2—C1—C6—C7	−171.95 (14)	C15—C16—C21—C20	173.97 (15)
C8—C1—C6—C7	9.0 (2)	C19—C20—C21—C16	−0.4 (3)
C2—C1—C8—C13	−121.05 (16)	C9—N2—C22—N1	3.3 (2)
C6—C1—C8—C13	58.0 (2)	C9—N2—C22—C23	177.90 (14)
C2—C1—C8—C9	54.0 (2)	C15—N1—C22—N2	−97.74 (18)
C6—C1—C8—C9	−126.86 (16)	C2—N1—C22—N2	74.85 (17)
C13—C8—C9—C10	−5.5 (2)	C15—N1—C22—C23	87.33 (17)
C1—C8—C9—C10	179.28 (15)	C2—N1—C22—C23	−100.07 (15)
C13—C8—C9—N2	175.05 (14)	N2—C22—C23—C24	−0.4 (2)
C1—C8—C9—N2	−0.1 (2)	N1—C22—C23—C24	174.33 (14)
C22—N2—C9—C10	132.30 (16)	N2—C22—C23—C28	−176.79 (16)
C22—N2—C9—C8	−48.3 (2)	N1—C22—C23—C28	−2.0 (2)
C8—C9—C10—C11	4.3 (2)	C28—C23—C24—C25	−0.7 (2)
N2—C9—C10—C11	−176.25 (15)	C22—C23—C24—C25	−177.15 (14)
C9—C10—C11—C12	−1.0 (3)	C23—C24—C25—C26	1.4 (2)
C10—C11—C12—C13	−0.9 (3)	C24—C25—C26—C27	−1.3 (3)
C11—C12—C13—C8	−0.4 (2)	C25—C26—C27—C28	0.4 (3)
C11—C12—C13—C14	174.18 (16)	C26—C27—C28—C23	0.4 (3)
C9—C8—C13—C12	3.6 (2)	C24—C23—C28—C27	−0.2 (2)
C1—C8—C13—C12	178.74 (15)	C22—C23—C28—C27	176.20 (16)

### Hydrogen-bond geometry (Å, °)

**Table:**

*D*—H···*A*	*D*—H	H···*A*	*D*···*A*	*D*—H···*A*
C3—H3···S1^i^	0.95	2.86	3.7216 (18)	152

Symmetry codes: (i) −*x*+2, *y*+1/2, −*z*+3/2.
